# The value of PLA2R antigen and IgG subclass staining relative to anti-PLA2R seropositivity in the differential diagnosis of membranous nephropathy

**DOI:** 10.1186/s12882-023-03273-4

**Published:** 2023-08-07

**Authors:** Dóra Bajcsi, László Bitó, Sándor Turkevi-Nagy, Tibor Nyári, Éva Kemény, Péter Légrády, György Ábrahám, Béla Iványi

**Affiliations:** Szent-Györgyi Albert Health Center, University of Szeged, Szeged, Hungary

**Keywords:** Anti-PLA2R antibodies, IgG subclass, IgG4-dominance, Membranous nephropathy, PLA2R antigen, Specificity

## Abstract

**Background:**

The diagnostic performance of PLA2R and IgG subclass staining of kidney biopsies relative to anti-PLA2R seropositivity in the differentiation of primary and secondary membranous nephropathy (pMN, sMN) was examined. Besides PLA2R staining – which has a lower specificity than anti-PLA2R antibody serology – there is insufficient knowledge to decide which IgG1-4 subtype immunohistological patterns (IgG4-dominance, IgG4-dominance/IgG1-IgG4-codominance or IgG4-dominance/IgG4-codominance with any IgG subtype) could be used to distinguish between pMN and sMN.

**Methods:**

87 consecutive Hungarian patients (84 Caucasians, 3 Romas) with the biopsy diagnosis of MN were classified clinically as pMN (n = 63) or sMN (n = 24). The PLA2R and IgG subclass staining was part of the diagnostic protocol. Anti-PLA2R antibodies were determined by an indirect immunofluorescence test in 74 patients with disease activity.

**Results:**

For pMN, the sensitivity of anti-PLA2R seropositivity was 61.1%, and the specificity was 90.0%; and similar values for PLA2R staining were 81.0%, and 66.7%, respectively. In all stages of pMN, IgG4-dominance was the dominant subclass pattern, while the second most frequent was IgG3/IgG4-codominance. The sensitivity and specificity scores were: IgG4-dominance 52.2% and 91.7%, IgG4-dominance/IgG3-IgG4-codominance 76.2% and 87.5%, IgG4-dominance/IgG1-IgG4-codominance 64.2% and 75%, and IgG4-dominance/codominance with any IgG subclass 92.1% and 70.8%, respectively. Anti-PLA2R seropositivity, glomerular PLA2R, and IgG4-dominance/codominance significantly correlated with each other. The IgG4 subclass was rarely encountered in sMN.

**Conclusion:**

In our series, IgG4-dominance had the highest specificity in the differentiation of MN, just as high as that for anti-PLA2R seropositivity. The specificity values of PLA2R staining and IgG4-dominance/codominance with any IgG subclass or IgG4-dominance/IgG1-IgG4 codominance were ≤ 75%. Apart from IgG4 dominance, IgG4-dominance/IgG3-IgG4-codominance also had good statistical value in differentiating pMN from sMN. As IgG subclass switching during the progression of pMN was not the feature of our cohort, pMN in Hungarian patients is presumed to be an IgG4-related disorder right from the start. Although anti-PLA2R seropositivity has become the cornerstone for diagnosing pMN, if a kidney biopsy evaluation is conducted, besides the staining of PLA2R antigen, the evaluation of IgG subclasses provides relevant information for a differential diagnosis. Even in cases with IgG4-dominance, however, malignancy should be thoroughly checked.

## Background

Membranous nephropathy (MN) is the most frequent cause of the nephrotic syndrome in non-diabetic adult patients worldwide [1], characterized morphologically by a thickening of the glomerular basement membrane (GBM) due to the presence of subepithelial immune deposits in glomerular capillary walls. The deposits undergo a series of changes of incorporation into the GBM, and this progression is described electron microscopically in four stages. MN is classified clinically as primary MN (pMN) or secondary MN (sMN). The clinical course of pMN ranges from spontaneous remissions to progressive disease.

pMN is responsible for 75–80% of cases of MN diagnosed in a kidney biopsy, and the vast majority are associated serologically with autoantibodies against a transmembrane glycoprotein of podocytes, the phospholipase A2 receptor (PLA2R) [2], discovered in 2009. The initial anti-PLA2R antibody response is directed against the immunodominant cysteine-rich domain of PLA2R protein, and then with epitope spreading, other domains, such as the C-type lectin domain1 and the C-type lectin domain7 are also targeted [3]. The predominant anti-PLA2R antibodies are of the IgG4 subclass, but other subclasses, such as IgG1 and IgG3, are present in lower amounts [2, 4]. During the evaluation of kidney biopsies, global and intense peripheral granular staining of polytypic IgG and C3, along with PLA2R antigen-positivity in a similar staining and distribution pattern indicate PLA2R-associated MN. In 2014, the second pathogenetic pathway of adult pMN, the axis of thrombospondin type-1 domain containing protein 7 A (THSD7A) autoantigen and anti-THSD7A antibodies was identified in anti-PLA2R-negative cases [5]. The PLA2R- and THSD7A-associated cases represent about 85% of pMN cases. Other MN subgroups were described quite recently, including the neural epidermal growth-factor-like 1 protein (NELL1)-associated MN [6, 7].

With sMN, it has an underlying disease association, such as systemic lupus erythematosus (SLE), infection, drug exposure or malignancy, and it is usually resolved once the cause is eliminated. Certain biopsy features are not typical for pMN, such as deposits positive for IgA, IgM, and C1q as in class V lupus nephritis, or the dominant IgG subclass is not IgG4, or the distribution of deposits is subglobal as in some cases with malignancy-associated MN [7, 8]. The absence of glomerular IgG4 and PLA2R [9], and glomerular leukocytosis [10] suggest malignancy-associated MN. In addition, enhanced glomerular staining of THSD7A [11] or NELL1 [7] may be observed in a fraction of patients with malignancy-associated MN.

The demonstration of circulating autoantibodies against PLA2R has become the standard process in the differentiation of pMN from sMN [12]. Nevertheless, some patients with apparent sMN might be positive for PLA2R antibodies, typically those with hepatitis B virus or C virus infection or sarcoidosis or malignancy [13]. It is not quite clear whether these patients have true sMN or the PLA2R-associated MN and the secondary disease is coincidental; and therefore, caution is required when trying to establish the clinical diagnosis of pMN solely on basis of PLA2R seropositivity, especially in patients with risk factors of neoplasia [14].

In the renal biopsy material of Ohio State University Medical Center (USA), early (stage 1) pMN tended to be IgG1-dominant and PLA2R-negative, and later stages (stages 2 to 4) tended to be IgG4-dominant and PLA2R-positive [8]. The change in IgG subclass dominance during disease progression was interpreted by the authors as a subclass switch in antibody response. Since IgG subclass staining is optional in the evaluation protocol of kidney biopsies, to the best of our knowledge only the Ohio State University Medical Center publications [8, 15], two studies from China [16, 17], and one from Japan [18] have covered the topic of IgG subtype distribution within the immune deposits of MN in native kidney samples. Here, we summarize our experiences acquired from the analysis of IgG subclass staining and PLA2R antigen staining in the diagnostic workup of MN in Hungarian patients.

## Patients and methods

*Patients.* Between 2011 and 2022, the evaluation of the renal biopsy samples of 87 adults led to the diagnosis of MN. The patients resided in the south-eastern part of Hungary. 84 were Caucasians, and 3 were Romas. A complete panel for autoimmune serology, hepatitis and HIV serology was performed to exclude SLE, other systemic autoimmune diseases and virus-associated MN. Malignancy was screened in each case *via* chest X-ray, abdominal ultrasound, gynecologyical/urological examinations (including a prostate specific antigen test), mammography, a fecal occult bleeding test and panendoscopic evaluation of the gastrointestinal tract in individuals above 40 years. After a thorough review of the patient’s medical history, drug-induced MN was also excluded. MN was assigned clinically to either pMN (n = 63; 72.4%) or sMN (n = 24; 27.6%). The median follow-up time was 56 months (range: 1 to 276 months). In the pMN group, 5 patients died for different reasons 1 to 8 months after the diagnosis of MN; systemic autoimmune disease or malignancy did not emerge. The patients’ demographic and clinical parameters are shown in Table 1.

**Table 1:** Clinical and laboratory data of patients with MN

Clinical data		pMN (n = 63)	sMN (n = 24)	P-value
	Age (years)	55.8 ± 14.3	52.8 ± 14.7	0.388
	Gender M/F	30/33	6/8	0.087
	BMI (kg/m^2^)	31.5 ± 6,3	25.2 ± 4.5	< 0.001
	SBP (mmHg)	131.4 ± 13.7	124.6 ± 14.8	0.046
	DBP (mmHg)	82.6 ± 8.5	78.8 ± 11.4	0.095
	HR (beat/min)	78.0 ± 10.9	74.0 ± 8.9	0.112
**Laboratory data**				
	Serum creatinine (µmol/l)	104.4 ± 63.3	98.0 ± 92.9	0.714
	CKD-G1 (number)	23	15	0.030
	CKD-G2 (number)	16	5	0.658
	CKD-G3 (number)	18	2	0.050
	CKD-G4 (number)	5	0	0.316
	CKD-G5 (number)	1	2	0.183
	eGFR-EPI (CKD G2-5) (ml/min./1.73 m^2^)	54.3 ± 20.8	50.8 ± 12.1	0.441
	serum albumin (g/l)	28.9 ± 7.2	30.0 ± 9.5	0.563
	serum cholesterol (mmol/l)	8.1 ± 2.8	7.3 ± 3.2	0.256
	serum trigliceride (mmol/l)	2.8 ± 2.7	2.1 ± 1.1	0.222
	proteinuria (g/day)	10.1 ± 6.0	5.2 ± 3.7	0.003
	nephrotic syndrome (number, %)	40 (63.5%)	10 (41.7%)	0.089
	nephrotic proteinuria without nephrotic syndrome (number, %)	19 (30.2%)	5 (20.8%)	0.435
	non-nephrotic proteinuria/ nephritic syndrome (number, %)	4 (6.3%)	9 (37.5%)	< 0.001

Values are in mean ± standard deviation (SD) where necessary

**Fig. 1:**
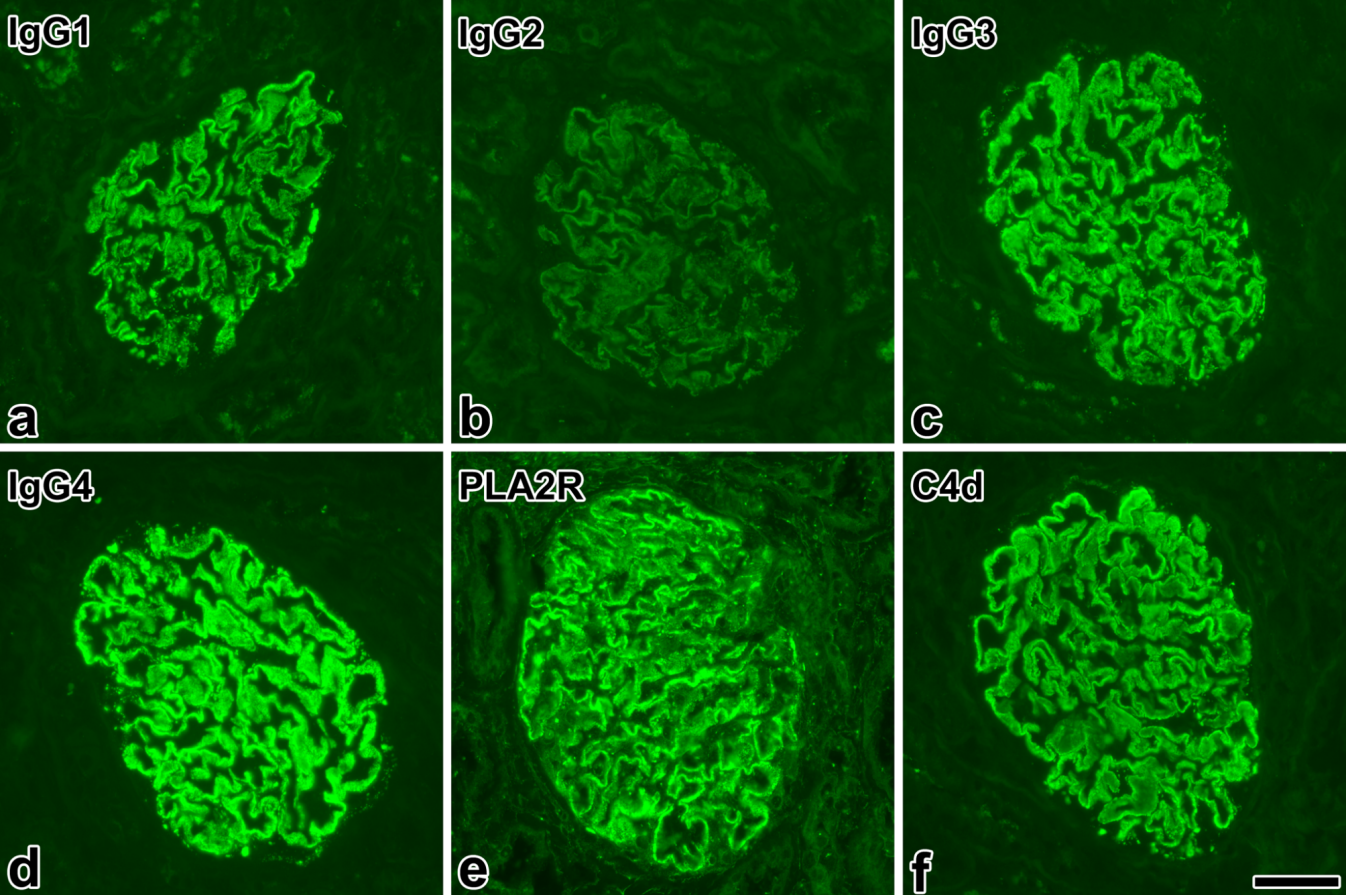
Typical expression of IgG subclasses, the PLA2R antigen and complement 4d in primary membranous nephropathy. Representative images of a case of PLA2R-positive, IgG4-dominant primary membranous nephropathy, stage 3. The staining intensity of IgG4 in immune deposits (d) was scored as 3+, and that of IgG1 (a) and IgG3 (c) as 2+, respectively. The reaction of IgG2 (b) was read as negative. The staining of the PLA2R antigen (e) in the deposits was bright. Note the complement 4d (C4d)-positivity of immune deposits (f), indicating complement activation *via* the lectin pathway. Immunofluorescence; images photographed at a magnification of 40x; the length of bar represents 50 μm

*Evaluation of kidney biopsies*. Samples obtained *via* an ultrasound-guided percutaneous kidney biopsy procedure were processed by standard techniques for light microscopy, direct immunofluorescence (IF) on frozen sections with FITC-conjugated antibodies against IgG, IgA, IgM, C3, C1q, kappa, lambda, and fibrinogen (Dako, Denmark; 1:20 dilution for IgG, and 1:10 dilution for the others), and electron microscopy. If granular peripheral IgG staining indicating MN was observed on IF, the case was further investigated with FITC-conjugated antibodies to IgG1-4 (The Binding Site, UK; 1:10 dilution) and PLA2R antigen (indirect IF method, Sigma-Aldrich, Switzerland; primary antibody dilution 1:10). The staining intensity was assessed and photographed at 10x objective lens magnification and graded semiquantitatively on a scale of 0 to 3 + (Fig. 1). IgG4-dominance was stated if the intensity score was higher by at least 1 level than that of the other IgG subclasses, and codominance was established if the intensity scores were similar among IgG subclasses. The electron microscopical reading of the stages were as follows: Stage 1: subepithelial deposits without basement membrane reaction; Stage 2: subepithelial deposits with basement membrane spikes between deposits; Stage 3: the spikes enclose the deposits and form a layer between the deposits and the podocytes; and Stage 4: electron-lucent areas in deposits become incorporated into the markedly and irregularly thickened GBM.

**Fig. 2:**
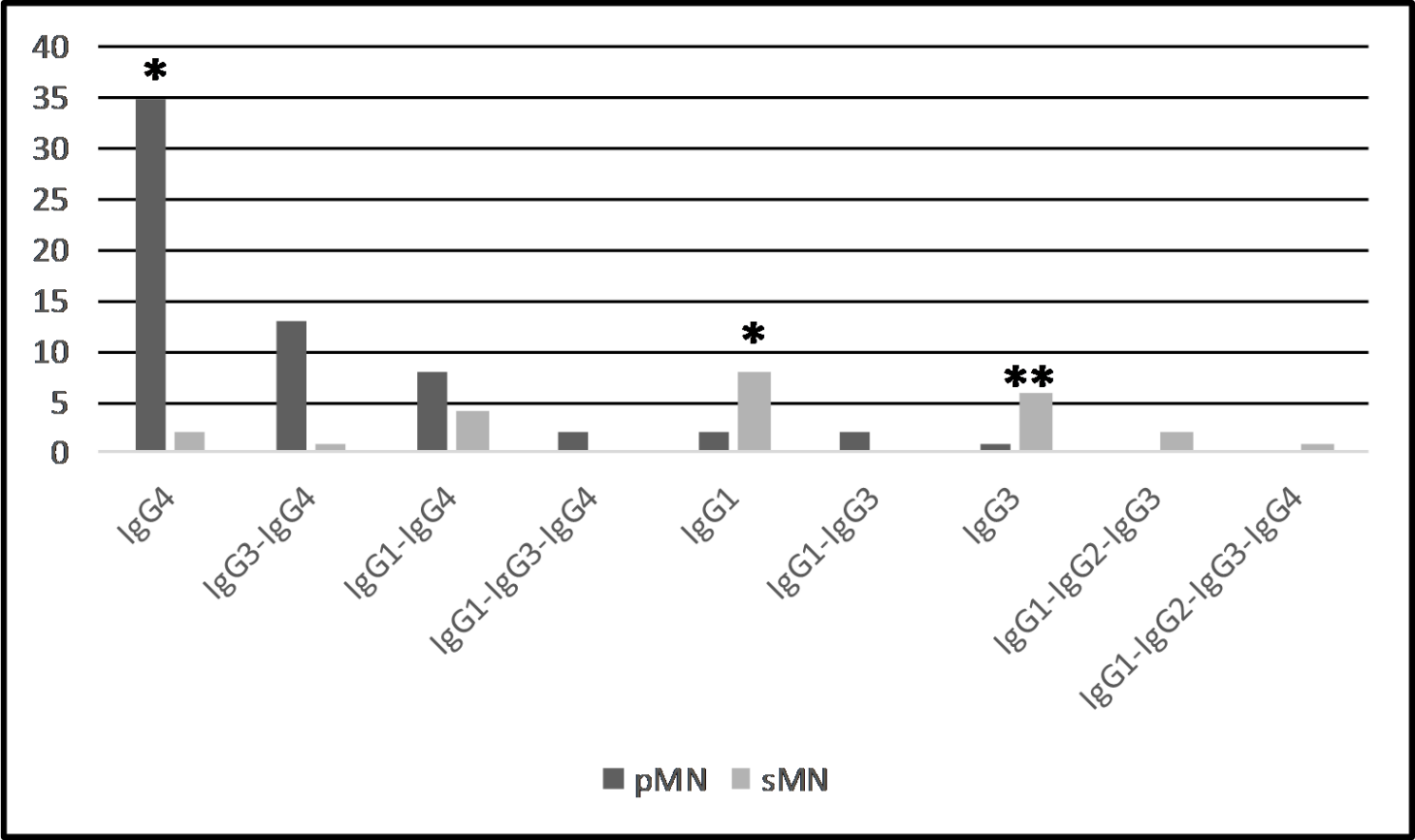
IgG subclass dominance/codominance in pMN and sMN. There is a significant difference in the prevalence of the IgG subtype dominance/codominance between cohorts of pMN and sMN (*p < 0.001, **p = 0.002, respectively)

*PLA2R seropositivity*. Blood serum samples were collected in 74 patients (54 pMN, 20 sMN) at the time of diagnosis (n = 69) or in the case of a relapse (n = 5). An indirect IF semiquantitative assay (Euroimmun US) was used to detect circulating anti-PLA2R antibodies. Serum samples were diluted at 1:10, and those found to be positive at 1:10 dilution were further diluted and evaluated. The results of the test given in this paper appear as either positive or negative.

**Fig. 3:**
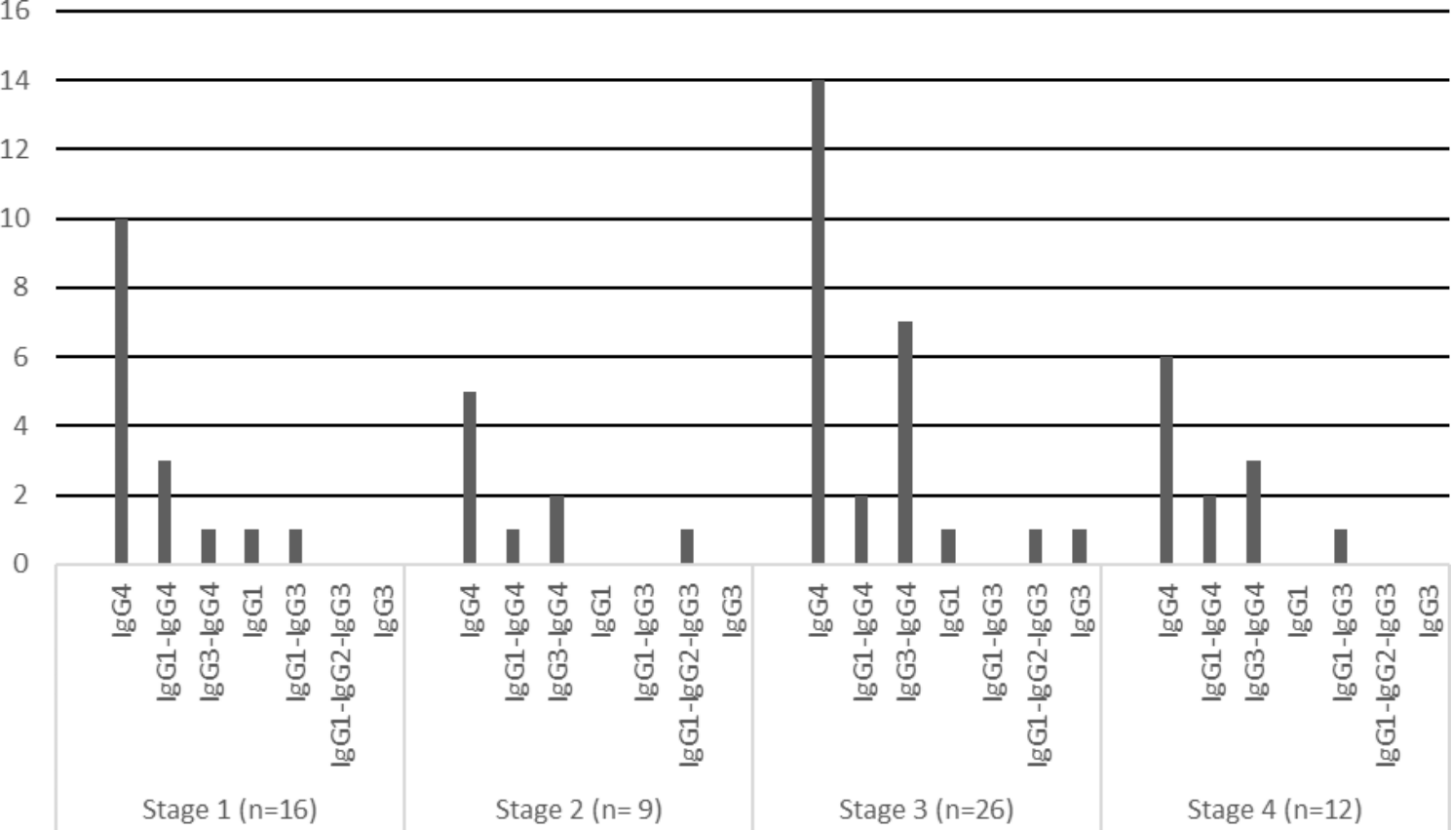
IgG subclass dominance/codominance in different stages of pMN

*Statistical analysis.* Continuous variables with a normal distribution were presented as the mean ± standard deviation and were compared using Student’s t-test. Categorical variables were described in percentage terms and the association between groups was analyzed using the Fisher exact test. A non-parametric Spearman rank correlation analysis was carried out to investigate the relationship among anti-PLA2R seropositivity, PLA2R antigen staining and patterns of IgG subclass variables. Also, the median follow-up time and its interquartile range were computed. A p value < 0.05 was treated as statistically significant.

**Fig. 4:**
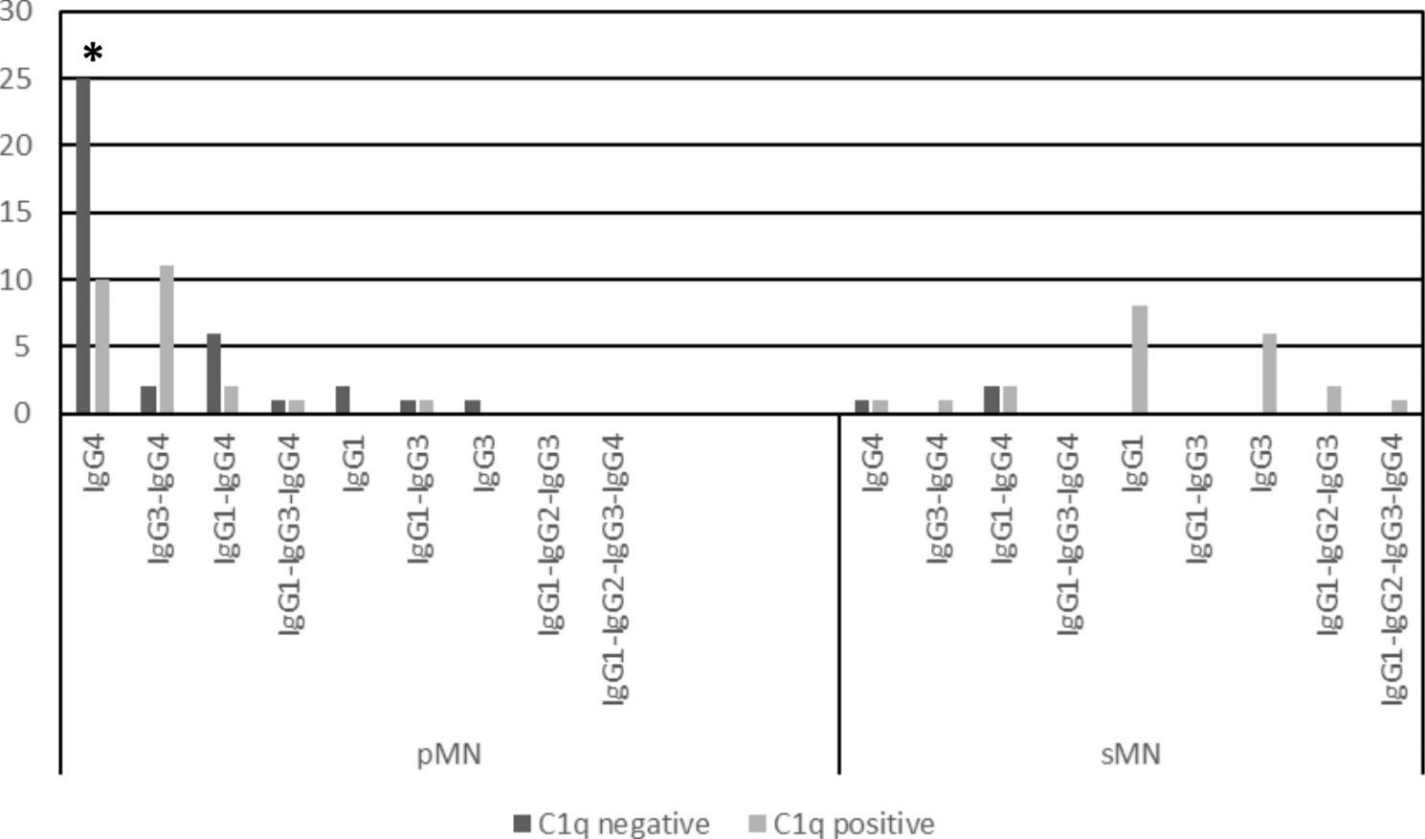
C1q positivity in pMN and sMN cases in the different IgG subtype dominance/codominance groups. Among the IgG4-dominant pMN cases, C1q negativity was more frequent than C1q positivity (*p=0.039)

## Results

Among patients assigned to pMN, 61.1% displayed anti-PLA2R seropositivity and 81.0% displayed PLA2R antigen staining positivity. 4 patients with PLA2R-negative pMN were tested for anti-thrombospondin 7 A-seropositivity, with a negative result (note: this test at our institution has been available since 2020).

In 3 patients out of the 24 placed in the sMN group (Table 2), the causal link between the glomerulopathy and the underlying disease was not entirely obvious. The first patient was a 57-year-old woman in whom T-cell acute lymphoblastic leukemia (T-ALL) and concurrent MN were diagnosed. The case was published recently elsewhere [19]. In brief, the proteinuria displayed a close relationship with the presence, chemotherapy-induced remission, and then relapse of T-ALL, thus it met the clinical criteria of malignancy-associated MN proposed by P. Ronco [20]. The pathological phenotype was characterized by mild and segmental positivity for glomerular PLA2R antigen, an incomplete global distribution of IgG1-IgG4 codominant deposits, stage 1–2 glomerulopathy, and more than 8 endocapillary leukocytes/glomerular profile. The immunostainings for glomerular THSD7A and NELL1 expression were negative. An indirect immunofluorescence assay for autoantibodies against PLA2R, THSD7A and neutral endopeptidase revealed a low anti-PLA2R titer. Although the leukemic lymphoblasts expressed CD10 (neutral endopeptidase) immunohistochemically, no change was observed in the CD10 expression of podocytes. It appeared that the T-ALL-associated MN was mediated by a strong autoimmune response for a hidden tumor antigen, and a weak one for PLA2R. The patient died six weeks after the renal biopsy procedure [19]. The second patient was a 66-year-old man, and he had PLA2R antigen-positive, IgG4-dominant, stage 3 MN, exhibiting glomerular leukocytosis; and anti-PLA2R antibodies were demonstrated in high titer. The nephrotic syndrome did not respond to glucocorticoid plus cyclophosphamide therapy. Metastatic squamous cell carcinoma of the oral cavity was diagnosed 12 months after the diagnosis of MN, and the patient passed away shortly thereafter. Glomerular leukocytosis was treated in retrospect as the feature of malignancy-associated MN [10], since thrombi in veins and/or glomeruli, dilation and congestion of glomerular and peritubular capillaries, disproportionate interstitial edema and/or focal interstitial hemorrhages suggestive of renal vein thrombosis were not observed in the biopsy specimen, and the ultrasound evaluation of the kidneys before and after the biopsy procedure did not raise the suspicion of renal vein thrombosis. A computed tomography angiography of kidneys, however, was not performed during the clinical evaluation of the patient. The third patient, a 53-year-old man with non-differentiated collagenosis (asymmetric oligoarthritis, gastrointestinal and other extrarenal symptoms, 0.73 g/day proteinuria, microhematuria, decreased renal function, raised inflammatory markers, negativity for ANCAs, positivity for anti-nuclear antibodies, anti-SSA antibodies, and anti-Jo1 antibodies, and mild positivity for rheumatoid factor-IgA) showed the simultaneous presence of stage 4 MN and crescentic IgA glomerulonephritis (probably IgA-vasculitis). The MN displayed IgG4-dominance and mildly intense positivity for PLA2R antigen.

**Table 2:** Number of pMN and sMN cases; causes of sMN

pMN (number, %)	63 (72.4%)
**sMN** (number, %)	24 (27.6%)
Lupus nephritis	16
Lupus-like nephritis*	1
HCV infection + SLE	1
SLE + rheumatoid arthritis + Sjögren syndrome	1
Ulcerative colitis	1
HBV infection + Graves-Basedow disease	1
Non-differentiated collagenosis, crescentic IgA glomerulonephritis (probably IgA-vasculitis) and MN	1
Malignancy**	2

*Renal limited disorder with “full house” positivity but without serological positivity and extrarenal manifestations of SLE

**Malignancy: squamous cell carcinoma of the oral cavity in a male patient, and acute T-cell leukemia in a female patient

For pMN, the sensitivity and specificity values for anti-PLA2R seropositivity, PLA2R antigen staining and patterns of IgG subclass distribution are shown in Table 3. As can be seen, the specificity of PLA2R seropositivity, IgG4-dominance, and IgG4-dominance/IgG3-IgG4-codominance was high, around 90%. Among the sensitivity values, IgG4-dominance/codominance with any other IgG subtype displayed the best sensitivity (92.1%), followed by PLA2R staining (81.0%), and IgG4-dominance/IgG3-IgG4-codominance (76.2%).

**Table 3:** Sensitivity and specificity values of serological and immunohistological examinations for the detection of pMN

	Sensitivity (%)	Specificity (%)
Anti-PLA2R seropositivity	61.1	90.0
PLA2R antigen staining	81.0	66.7
IgG4-dominance	52.2	91.7
IgG4-dominance/codominance with any other IgG subtype	92.1	70.8
IgG4-dominance/IgG1-IgG4-codominance	64.2	75.0
IgG4-dominance/IgG3-IgG4 codominance	76.2	87.5

Regarding the distribution of IgG subclasses in pMN (Fig. 2), IgG4-dominance was the predominant pattern (55.5%). The second most frequent pattern was IgG3/IgG4-codominance (20.6%), and the third was IgG1/IgG4-codominance (14.2%). In contrast, IgG1-dominance, followed by IgG3-dominance characterized sMN. IgG4-dominance was rarely encountered, observed in the case of MN and concurrent crescentic IgA glomerulonephritis, and in the case of MN and squamous cell carcinoma of the tonsils. Of interest, PLA2R antigen staining positivity was detected in 3 out of the 5 non-lupus sMN cases.

The analysis of correlation among anti-PLA2R seropositivity, PLA2R antigen staining, and patterns of IgG subclass distribution (Table 4) found that almost all the parameters had a significant correlation with each other; that is, anti-PLA2R seropositivity had an excellent correlation with PLA2R antigen staining, IgG4-dominance/codominance, and IgG4-dominance/IgG3-IgG4-codominance, respectively. Anti-PLA2R seropositivity and PLA2R antigen staining were concordantly positive in 29 and concordantly negative in 7 out of the 54 pMN cases. Anti-PLA2R seropositivity was accompanied by negative PLA2R staining in 4 patients; and conversely, positive PLA2R staining was accompanied by a negative anti-PLA2R antibody test result in 14 patients out of the 54 pMN cases. Most of the latter cases were classified electron microscopically as early MN because stage 1 was observed in 8 samples, and in 2 cases with stage 4 disease fresh subepithelial deposits were noted, indicating a relapse in the formation of immune deposits. There were 5 cases with positive PLA2R antigen staining that did not display IgG4-dominance/codominance, and there were 12 cases in which IgG4-dominance/codominance was accompanied by negative PLA2R staining.

**Table 4:** Correlation between the immunohistological parameters and anti-PLA2R seropositivity

Examinations		p value of correlation
Anti-PLA2R seropositivity	PLA2R antigen staining	< **0.001**
Anti-PLA2R seropositivity	IgG4-dominance	0.057 (NS)
Anti-PLA2R seropositivity	IgG4-dominance/codominance	**< 0.001**
Anti-PLA2R seropositivity	IgG4-dominance/IgG1-IgG4-codominance	0.108 (NS)
Anti-PLA2R seropositivity	IgG4-dominance/IgG3-IgG4-codominance	**< 0.001**
PLA2R antigen staining	IgG4-dominance	0.005
PLA2R antigen staining	IgG4-dominance/codominance	0.011
PLA2R antigen staining	IgG4-dominance/IgG1-IgG4-codominance	0.007
PLA2R antigen staining	IgG4-dominance/IgG3-IgG4-dominance	0.005

Correlations were analyzed in 74 cases

NS: not significant

Regarding the distribution pattern of IgG subclasses in different stages of pMN, IgG4-dominance was observed in all the stages of pMN (Fig. 3). There was no significant difference between the prevalence of IgG4-dominance and IgG4-dominance/codominance among the stages (p = 0.950, p = 0.849).

C1q positivity was significantly more frequent in sMN than pMN (p < 0.001). In pMN, C1q positivity was mainly associated with cases of IgG3-IgG4-codominance (Fig. 4.). Among IgG4-dominant pMN cases, C1q negativity was significantly more frequent than C1q positivity.

## Discussion

In the clinical management of patients with MN, it is mandatory to exclude SLE, infections, drugs and malignancy, regardless of whether anti-PLA2R antibodies and/or anti-THSD7A antibodies are absent or present [12]. However, the intensification of diagnostic procedures for looking for a secondary origin may vary in different nephrology units. To look for malignancy, some centers conduct the basic examinations of chest X-ray, abdominal ultrasound, a pelvic/rectal exam for gynecological/urological malignancy, the fecal occult bleeding test, and mammography, while others perform panendoscopy of the gastrointestinal tract. Still it is possible that the malignancy may only be detectable on a PET or CT scan or it cannot be detected at the time of diagnosis of MN, just several months later [21]. Moreover, it is not clear how intensively and how often clinicians should carry out the examinations for sMN. And, if a potentially secondary origin is identified, it may not be easy to determine the causal relationship between the underlying disease and MN; an association of the two disorders may be merely coincidental. The results of PLA2R antigen and IgG subclass distribution in glomerular deposits [13, 22] can provide relevant information alongside clinical examinations in the separation of pMN from sMN. This is discussed below.

The features of pMN presented refer to patients living in an agricultural region of Central-Eastern Europe, and their being almost exclusively Caucasians. As far as we know, no similar study has been published for this part of Europe. The diagnostic performance of PLA2R staining relative to anti-PLA2R seropositivity for the detection of pMN was 81% sensitivity and 66.7% specificity for glomerular PLA2R, and 61.1% sensitivity and 90% specificity for anti-PLA2R seropositivity. In the cohort of 42 pMN patients from France [23] there were 10 patients with positive PLA2R staining and no measurable antibodies in the serum, and 3 patients with high levels of anti-PLA2R antibodies and negative PLA2R glomerular staining (biopsy sensitivity 74%; serum sensitivity 57%). In the study of 22 pMN from Japan, glomerular PLA2R deposits were observed in 64% of cases, and serum anti-PLA2R antibodies in 55% of cases [18]. In the meta-analysis of 19 studies involving 1160 patients PLA2R staining positivity had a sensitivity of 78% and specificity of 91% for detecting PMN, while anti-PLA2R1 seropositivity had a sensitivity of 68% and specificity of 97% for the same set of patients [24]. These values are slightly different from those in the present study. Several factors might influence the diagnostic accuracy of glomerular PLA2R staining and anti-PLA2R seropositivity for pMN detection, such as differences in the sensitivity of the technique used for the testing of anti-PLA2R antibodies [4, 25], the method of immunostaining [26], the test time interval [27] and the dynamics of antibody response during the progression of MN. Anti-PLA2R antibodies may already be present in the non-nephrotic stage of MN [28], and conversely, they may be absent at the onset of the nephrotic stage of the disease [4, 29]. Seronegative pMN with glomerular PLA2R-positivity may turn into seropositive pMN provided the follow-up is sufficiently long [4, 29, 30]. *Taking these factors in account, glomerular PLA2R staining usually has better sensitivity, and anti-PLA2R seropositivity usually has a higher specificity score for pMN detection.*

The discordance between the results of anti-PLA2R serology and PLA2R staining may be due to the following: (1) The patient develops a spontaneous serological remission or has an undulating serum level of anti-PLA2R antibody, while the PLA2R antigen is still demonstrable in glomerular immune deposits [27]. (2) According to the hypothesis of the kidney as a sink, the anti-PLA2R antibodies may sometimes appear in the blood after the kidneys’ buffering capacity has been exceeded [27, 30, 31]. This may be so in majority of cases in our series, since among the 14 seronegative cases with glomerular PLA2R positivity, 8 cases were in stage 1 disease, and 2 cases with stage 4 disease had fresh subepithelial deposits. (3) Anti-PLA2R serological positivity with lack of staining on biopsy rarely occurs; as indirect IF has a higher sensitivity but lower specificity in detecting anti-PLA2R antibodies. An ELISA assay could exclude false positive results, but the assay was not available in Hungary during the study period.

In 2016, investigators from the USA [22] and China [16] independently found IgG subclass staining to be a valuable tool in the differentiation of pMN from sMN: IgG4-dominance/codominance characterized pMN in both studies. Our study on Hungarian patients provides similar observations. It remains unclear, however, whether IgG4-dominance or IgG4-dominance/codominance with any other IgG subclass or IgG4-dominance/IgG1-IgG4-codominance has the highest specificity for the detection of pMN. In our cohort, IgG4-dominance performed the best with 91.7% specificity, comparable with the performance of anti-PLA2R seropositivity (90.0%) and much higher than the specificity of glomerular PLA2R positivity (66.7%). The second and the third subclass distribution pattern was IgG3/IgG4-codominance, and IgG1/IgG4-codominance, respectively; the diagnostic performance of IgG4-dominance/IgG3-IgG4-codominance indicated good sensitivity (76.2%) and high specificity (87.5%). In the cohort of 286 pMN cases from Columbus, USA, IgG1 (97%) was the most frequent subclass, followed by IgG4 (94%), and the staining intensity assessed semiquantitatively was 1.9 for IgG1, and 2.4 for IgG4, resulting in an IgG1/IgG4-codominant or an IgG4-dominant staining pattern [22]. The IgG subclass distribution pattern in our patients significantly differed from that in the Columbus series, because *we observed a striking IgG4-dominance in all stages of pMN*, followed by IgG4-dominance/IgG3-IgG4 codominance distribution pattern. Consistent with our findings, the IgG4 subclass dominated all the stages of PLA2R-associated MN in patients from Beijing, China [17]. In the series from the Mayo Clinic (USA), analyzing the temporal IgG subtype changes in recurrent idiopathic MN, IgG4 was the dominant IgG subtype, regardless of the time from recurrence or PLA2R-association, and in the majority of recurrent MN the (co)dominant subtype did not change over time [32]. While we cannot provide a reasonable explanation for the discordant observations, it appears that *pMN is a predominantly IgG4-related disorder right from the start in Hungarian patients*. One of our reviewers had the opinion that this conclusion is overstated without investigating repeat biopsies or measurements of IgG1 and IgG4 antibodies in patients’ serum. Indeed, these results would be of value here, but the clinical situation did not necessitate repeating the renal biopsy procedure and/or a subclass-specific analysis of anti-PLA2R antibodies. Probably for similar reasons, however, none of the publications of Huang et al. from Columbus, USA [8], Dong et al. [16] and Cui et al. [17] from China, and Hayashi et al. [18] from Japan included the results of repeat biopsies or a subclass-specific analysis of anti-PLA2R antibodies while investigating the presence or absence of an IgG subclass switch from IgG1 to IgG4 in pMN. *In summary, an IgG subtype analysis and a glomerular PLA2R staining support the differentiation of pMN from sMN, and IgG4 subclass dominance has the highest specificity score in our cohort.*

In agreement with the IgG subclass distribution pattern of pMN, C1q-positivity was obviously not characteristic for pMN in our cohort, since IgG4 cannot bind C1q, a major precursor in the classical pathway. The lectin pathway might be involved in complement activation in pMN [33], activated for example by aberrantly glycosylated IgG4 [34], because C4d, a product of mannose binding lectin-associated serine proteases is commonly found in pMN (see Fig. 1). The alternative pathway does not cleave C4.

Malignancy-associated MN is a challenging subset of MN. Pathologically, an increased number of leukocytes in glomerular capillaries [10], the dominance of IgG1 and IgG2 subclasses in deposits [9, 35], the negativity of deposits for PLA2R and subglobally distributed deposits [7] all suggest that the patient has a malignant tumor, often clinically hidden. Malignancy is usually found within a year of the diagnosis of MN [21], and in most cases it is discovered before or at the time of the diagnosis of renal disease. Solid tumors, such as carcinomas of the lungs, prostate, gastrointestinal tract, breast, kidney, urinary bladder or the skin may have an association with MN [36], but it is difficult to prove a link between the two disorders. In 1999, P. Ronco proposed three criteria for diagnosing malignancy-associated MN: the treatment-induced remission of cancer is followed by the remission of the nephrotic syndrome; the recurrence of the neoplasia is accompanied by a renal relapse; and tumor antigens and antitumor antibodies are detected within the subepithelial deposits [20]. However, the tumor antigen frequently remains undetected during the management of the patients. Although malignancy-associated MN is usually seronegative for anti-PLA2R antibodies [9, 14], there are occasionally cases which display a high titer of anti-PLA2R antibodies [37, 38]. Therefore, some authors think that the subtype analysis of glomerular IgG, along with the summarized biopsy features of malignancy-associated MN is not sufficient to provide a definite diagnosis of pMN or sMN [11, 39]. If malignancy-associated MN is suspected, the glomerular antigen status should be completed with THSD7A and NELL1 examinations [7, 40]. The evaluation of NELL1 axis is, however, not yet widely available, and we also performed it in an international collaboration.

In our series, the clinicopathological features of T-ALL and concurrent MN negative for THSD7A and NELL1, and segmentally and mildly positive for PLA2R indicated true paraneoplastic MN, although the exact pathogenetic link between the leukemia and MN remained unexplored. In the case of a 66-year-old man with PLA2R antigen-positive, IgG4-dominant, stage 3 MN and glomerular leukocytosis, and metastatic squamous cell carcinoma of the oral cavity one year after the diagnosis of MN, the results obtained were not enough to conclude whether the patient had PLA2R-associated pMN with coincidentally discovered malignancy, or the malignancy induced the formation of anti-PLA2R autoantibodies that resulted in MN. Since the proteinuria did not respond to conventional therapy of MN, we felt that the case should be placed in the sMN group. Lastly, we had the case of a 53-year-old man with non-differentiated collagenosis in whom concurrent crescentic IgA glomerulonephritis and stage 4 IgG4-dominant MN with mildly intense positivity of PLA2R antigen were found. Because of the features of non-differentiated collagenosis, the case was included in the sMN group, although it might just represent the coincidence of two primary renal diseases. If we assigned the last two patients erroneously, and they were clear examples of pMN, then *IgG4-dominance was exclusively a feature of pMN in Hungarian patients*.

The KDIGO 2021 Clinical Practice Guideline for the Management of Glomerular Diseases [12] stated that in the case of anti-PLA2R seropositivity, normal eGFR and no immunosuppressive therapy, carrying out a kidney biopsy is not needed for making the clinical diagnosis of MN. If immunosuppressive therapy needs to be administered, a kidney biopsy should be considered. In contrast, the biopsy procedure should be conducted if there is an unusual clinical course or a rapid eGFR decrease is detected or there are serological abnormalities (for instance positive nuclear antibodies) or there is unresponsiveness to immunosuppressive therapy [12]. According to the review of Ronco [41] based on the study of Bobart el al [42]., a biopsy procedure is not recommended if eGFR > 60 ml/min/1.73 m^2^, if anti-PLA2R serology is positive and there is no evidence of a secondary cause or diabetes. Among the serologically evaluated 74 MN patients, 6 patients had anti-PLA2R positivity and normal eGFR (> 90 ml/min./m^2^). However, immunosuppressive treatment was initiated in all of them, so on basis of the KDIGO protocol [12] they were placed in the group where a biopsy should be considered. According to Ronco’s recommendation [41], the biopsy confirmation of MN could in retrospect have been replaced by a serology-based diagnostic approach in 10 patients out of the 74 patients, and this indicates that *the diagnosis of MN still necessitates the evaluation of the kidney biopsy sample in the majority of patients*. The good point of our study was that we compared the statistical value of widely available immunohistological methods in the differentiation of pMN and sMN. Previously there was no direct comparison of the specificity of IgG4-dominance and different IgG4-dominance/codominance patterns and there was little knowledge about the comparison of PLA2R-based diagnostics (serology, immunohistology) and IgG1-4 subtype evaluation. The limitations were that most sMN cases were lupus MN, the number of malignancy-associated MN was low, and the antigen status was not always complete.

In conclusion, in the retrospective cohort of MN patients from Central-Eastern Europe, anti-PLA2R serology had a lower sensitivity score for pMN than that given in most of the publications; however the specificity of seropositivity was comparably high, hence it has a definite value in the classification of MN. On reevaluating the biopsy indication of our cases on the basis of new non-invasive approaches, the biopsy procedure had to be performed or it should have been considered in most of the cases. With a histological evaluation, PLA2R staining alone had a lower specificity score than that for serology. Nevertheless, we recommend that it be supplemented with an IgG subtype analysis, since IgG4-dominance, followed by IgG4-dominance/IgG3-IgG4-codominance offers high specificity in differentiating pMN from sMN. However, malignancy should still be carefully looked for. The possibility of an IgG1 to IgG4 subtype switch during the progression of pMN was not demonstrated in the Hungarian cohort.

The classification of MN into primary and secondary is on the way to being replaced by the antigen detected [43], because the presence of autoantibodies is not always in accord with the clinical definitions of pMN and sMN. In clinical practice, however, the results of anti-PLA2R serology, PLA2R immunohistology, along with the determination of IgG subclass in glomerular immune deposits are widely available, and their results might narrow the range of tests required for evaluating MN patients.

## Data Availability

The datasets used and/or analyzed during the current study are available from the corresponding author on reasonable request.

## List of abbreviations

GBM: Glomerular basement membrane
IF: Immunofluorescence
Jo-1 antibody: Myositis specific autoantibody directed against the histidyl-tRNA synthetase
MN: Membranous nephropathy
NELL1: Neural epidermal growth-factor-like 1 protein
PLA2R: Phospholipase A2 receptor
pMN: Primary membranous nephropathy
sMN: Secondary membranous nephropathy
SLE: Systemic lupus erythematosus
SSA: Sjögren’s-syndrome-related antigen A
T-ALL: T-cell acute lymphoblastic leukemia
THSD7A: Thrombospondin type-1 domain containing protein 7 A
